# Perceived organizational silence climate and turnover intention: indirect associations through learned helplessness, psychological withdrawal, and job burnout

**DOI:** 10.3389/fpsyg.2026.1962689

**Published:** 2026-10-05

**Authors:** Wooik Shin, Boyoung Kim

**Affiliations:** Seoul Business School, aSSIST University, Seoul, Republic of Korea

**Keywords:** job burnout, learned helplessness, perceived organizational silence climate, psychological withdrawal, turnover intention

## Abstract

This study analyzed how the perceived organizational silence climate held by members of digital platform service organizations is related to turnover intention through learned helplessness, psychological withdrawal, and job burnout. The perceived organizational silence climate is defined here as an individual-level psychological climate perception: the degree to which an individual employee perceives that a belief is prevalent within the organization that raising problems or expressing opinions is of no use or may lead to disadvantages. A cross-sectional online survey was conducted with 346 employees working at digital platform service firms in South Korea. After the validity of the measurement model was examined through confirmatory factor analysis, the relationships among the variables were analyzed using latent-variable structural equation modeling, and the model-implied indirect associations were estimated with bias-corrected bootstrap confidence intervals based on 5,000 resamples. The results showed that the perceived organizational silence climate was positively associated with learned helplessness, psychological withdrawal, and job burnout. All three variables were also positively associated with turnover intention. Each model-implied indirect association through learned helplessness, psychological withdrawal, and job burnout was statistically significant, whereas the direct association was not. Pairwise comparisons of the three specific indirect associations were not statistically significant, indicating that the three pathways could not be distinguished in magnitude. These findings extend the focus of organizational silence research from individual silence behavior to employees’ perception of the organizational silence climate and test, within a single parallel mediation model, the cognitive, behavioral-relational, and affective pathways that connect the organizational silence climate to turnover intention. The findings further suggest that, to manage turnover intention, it is necessary to diagnose employees’ perceptions of both the futility and the riskiness of voice and to improve organizational environments in which the usefulness of expressing opinions is perceived to be low or the possibility of disadvantage is perceived to be high. The data are cross-sectional and were collected from a single source. The estimates reported here therefore describe associations rather than causal effects, and the model-implied indirect associations require confirmation in longitudinal or multiwave designs.

## Introduction

Employees sometimes recognize problems at work but do not voice their opinions or share information. This phenomenon is central to research on organizational decision-making and job attitudes. Morrison and Milliken (2000) viewed organizational silence as an organization-level phenomenon in which a majority of employees withhold important opinions and information rather than sharing them. Where silence persists, problems are less likely to be surfaced in a timely manner, and organizational learning and change may be constrained. Recent research regards silence not as the mere absence of voice but as a phenomenon that is associated with employees’ psychological resources and job attitudes in ways that are distinct from voice (Sherf et al., 2021; Morrison, 2023). Evidence also shows that silence and voice are associated with group- and organization-level contexts, and that voice is linked to individual- and organization-level outcomes (Tangirala and Ramanujam, 2008; Morrison et al., 2011; Bashshur and Oc, 2015).

Research on organizational silence has increasingly distinguished among its motives and forms. Van Dyne et al. (2003) divided silence into acquiescent silence, in which the possibility of change has been abandoned, defensive silence, which is intended to avoid disadvantage, and prosocial silence, which is intended to protect others. Knoll and van Dick (2013) added opportunistic silence, and Brinsfield (2013) presented a range of silence motives including resignation, fear, and relationship protection. More recently, attention has expanded beyond silence behavior to the organizational context in which voice comes to be perceived as meaningless or risky (Lotfi Dehkharghani et al., 2023; Morrison, 2023). Implicit beliefs about the risks and benefits of voice, psychological safety, and voice efficacy are key conditions that determine whether employees speak up (Detert and Edmondson, 2011; Liang et al., 2012; Edmondson and Lei, 2014; Frazier et al., 2017).

When employees perceive the voice environment of their organization as silent, they may experience a sense of helplessness in that it is difficult to change the situation through their own efforts (Abramson et al., 1978; Morrison and Milliken, 2000; Chung et al., 2017), and they may exhibit psychological withdrawal by retracting their expectations of and emotional investment in the organization (Kahn, 1990; Milliken et al., 2003; Golden and Veiga, 2018). Repeated self-censorship and emotion suppression may consume psychological resources and are also related to job burnout. In fact, silence has been positively related to job burnout, and a significant association between the two phenomena has also been confirmed in meta-analyses (Sherf et al., 2021; Parker et al., 2025; Lainidi et al., 2026). Holland et al. (2026) reported that employee silence mediated the associations of workload with turnover intention and work engagement, and that trust in management moderated the workload–silence association. Prior studies have linked negative organizational environments to turnover intention through distinct psychological processes (Cicek et al., 2021; Hung et al., 2025; Zhang et al., 2025; Katıtaş, 2026). Guanxi-based human resource management practices were positively associated with turnover intention through reduced work meaningfulness (Zhang et al., 2025), and workload and emotional demands were positively associated with turnover intention through job burnout (Hung et al., 2025), whereas organizational silence was linked to turnover intention through work alienation (Katıtaş, 2026) and organizational cynicism was linked to turnover intention through organizational support (Cicek et al., 2021). These relationships need to be examined more concretely in digital platform service organizations, where the sharing of frontline knowledge and opinions is important for service improvement.

Digital platform service organizations continuously revise their services and operating methods in response to user reactions and technological change, and service innovation is achieved through collaboration across diverse jobs (Nambisan et al., 2017; de Reuver et al., 2018). Algorithm-based management and digital work design can increase the efficiency of work coordination while at the same time altering employees’ experiences of control, autonomy, and agency (Kellogg et al., 2020; Duggan et al., 2020; Curchod et al., 2020; Parent-Rocheleau and Parker, 2022). In such environments, the need to share frontline knowledge and improvement suggestions quickly increases, but when the usefulness of voice is perceived to be low and its risk high, employees may choose silence and psychological withdrawal instead of information sharing. Related prior studies have shown that quiet quitting is related to turnover intention through job satisfaction and affective commitment (Kim and Sohn, 2024), that destructive leadership is positively associated with turnover intention through job burnout (Chen et al., 2025), and that psychological safety is involved in shifts between voice and silence in digital virtual teams (Dong and Xing, 2026). These studies highlight the importance of psychological withdrawal and turnover in digital environments, but do not examine the organizational silence climate together with different psychological pathways.

Existing research has specified the forms and motives of organizational silence and has clarified the relationships of silence with job burnout and turnover intention. However, an integrative literature review pointed out that the mixed measurement of silence and voice makes it necessary to distinguish the constructs (Lainidi et al., 2023), and research on service occupations has focused on psychological safety and voice behavior (Hu et al., 2024). Three research gaps remain. First, prior research has focused mainly on individual silence behavior and its motives (Brinsfield, 2013; Lotfi Dehkharghani et al., 2023), and evidence on the relationship between employees’ perception of a silent organizational voice environment and turnover intention remains limited. Second, related studies have verified specific mediating variables such as work alienation or job burnout individually, or have examined moderating factors such as trust in management (Katıtaş, 2026; Holland et al., 2026), and evidence that simultaneously tests learned helplessness, psychological withdrawal, and job burnout within a single model remains scarce. Third, although the relationship between psychological withdrawal and turnover intention has been reported in digital transformation environments (Kim and Sohn, 2024), evidence on the relationship between the perceived organizational silence climate and turnover intention through distinct psychological pathways in digital platform service organizations remains limited. To address these gaps, this study examines the association between the perceived organizational silence climate and turnover intention, together with the indirect associations transmitted through learned helplessness, psychological withdrawal, and job burnout, among employees of digital platform service organizations, and presents implications for the voice environment and employee management.

## Theoretical background and hypotheses

### The perceived organizational silence climate from a job demands-resources perspective

Job demands-resources theory explains the process through which the perceived organizational silence climate is connected to negative psychological states. This theory distinguishes between job demands, which require sustained effort and induce strain, and job resources, which support goal attainment, learning, and growth. When job demands are high and job resources are insufficient, burnout and negative outcomes may emerge through a health impairment process (Demerouti et al., 2001; Schaufeli and Bakker, 2004). Subsequent theoretical reviews and a meta-analysis synthesizing multiple longitudinal studies also supported the health impairment process, in which higher job demands and insufficient job resources lead to burnout and negative job outcomes (Bakker and Demerouti, 2007; Lesener et al., 2019). According to conservation of resources theory, individuals seek to protect resources such as a sense of control, emotional energy, and relational investment, and resource loss leads to strain and withdrawal (Hobfoll, 1989). A situation in which voice is perceived as meaningless or risky prevents employees from expressing problems even when they recognize them, thereby requiring self-censorship and emotion suppression. It also weakens employees’ sense that their judgment can influence decision-making and can therefore be regarded as a job demand that imposes a psychological burden (Morrison and Milliken, 2000; Sherf et al., 2021). Accordingly, the perceived organizational silence climate focuses on expectations about the acceptability of voice and about the consequences that follow voice, rather than on actual silence behavior. When experiences of appraising the usefulness of voice as low and the possibility of disadvantage as high are repeated, employees must continuously regulate their own judgments and emotions. In this process, a sense of control, relational investment, and emotional energy are weakened, and this may manifest as learned helplessness, psychological withdrawal, and job burnout. The findings that silence is related to burnout in ways distinct from voice (Sherf et al., 2021) and the longitudinal evidence that a voice climate reduces subsequent silence (Parker et al., 2025) support this interpretation.

Morrison and Milliken (2000) theorized organizational silence as an organization-level phenomenon in which a majority of employees do not speak up about important information and opinions. When managers interpret negative feedback as a threat to the organization, or assume that employees prioritize their own interests over those of the organization, they may distrust employees’ opinions and structurally restrict opportunities for voice. In a qualitative study, Milliken et al. (2003) identified concerns about negative labeling, damaged relationships, and weakened influence as reasons for employee silence. These findings show that silence can arise not only from individual dispositions but also from repeated managerial practices and expectations about the consequences of voice. The managerial practices most often implicated are abusive or dismissive supervisory behavior, the routine failure to act on or respond to the suggestions that employees do raise, the absence of formal and informal channels through which concerns can be reported, the treatment of dissent as disloyalty in evaluation and promotion decisions, and top-down decision-making in which negative feedback is interpreted as a threat rather than as information (Morrison and Milliken, 2000; Milliken et al., 2003; Vakola and Bouradas, 2005; Detert and Edmondson, 2011; Xu et al., 2015; Harlos, 2016). If organizational silence is an organization-level phenomenon, the perceived organizational silence climate is an individual-level cognition in which employees interpret the voice environment. A psychological climate perceived by an individual is the result of evaluating the work environment at the individual level, and it can be treated as an organization-level climate when sufficient agreement among employees is demonstrated (James et al., 2008). In this study, the perceived organizational silence climate refers to the degree to which an individual employee perceives two beliefs to be prevalent within the organization. The first is the belief that nothing will change even if one speaks up, which is a perception of futility. The second is the belief that speaking up may lead to disadvantage, which is a perception of risk. The construct therefore refers neither to the shared climate itself nor to an individual’s actual silence behavior. These two perceptions are, respectively, linked to the motives of acquiescent and defensive silence (Van Dyne et al., 2003; Knoll and van Dick, 2013), but the focus of this study lies in the voice environment that gives rise to such judgments rather than in the types of silence motives. Two clarifications follow from this distinction. First, the construct examined here is a psychological climate in the sense of James et al. (2008): it is an individual employee’s appraisal of the voice environment, and it is measured, modeled, and interpreted exclusively at the individual level. Organizational climate in the strict sense is an aggregate property whose existence must be demonstrated through within-unit agreement, conventionally using *r*_wg_, ICC(1), and ICC(2) (James et al., 2008; Schneider et al., 2013). No such aggregate-level claim is made in this study, and no aggregate-level parameter is estimated. The qualifier perceived is therefore not ornamental: it marks the construct as a perception held by an individual respondent. Second, the referent of that perception is nevertheless the organization rather than the respondent’s own conduct, which is why the label retains organizational silence climate. This distinguishes the construct from individual silence behavior, which concerns what an employee does, and from organizational silence in the sense of Morrison and Milliken (2000), which is a property of the organization itself. Table 1 states the level of analysis of each of these concepts explicitly. The attitudes of top management and supervisors and the availability of formal and informal communication opportunities can serve as cues through which employees interpret the possibility of voice in the organization (Vakola and Bouradas, 2005).

**Table 1:** Distinctions between the perceived organizational silence climate and related concepts.

Concept	Core focus	Level of analysis	Prior research
Individual silence behavior	The behavior of an individual employee withholding opinions, information, and concerns about work-related problems	Individual (behavior)	Van Dyne et al. (2003); Brinsfield (2013)
Voice	The behavior of voluntarily expressing work-related ideas and suggestions for the purpose of organizational improvement	Individual (behavior)	Van Dyne et al. (2003); Morrison (2023)
Organizational silence	An organization-level phenomenon of the collective withholding of important information, shaped by structural conditions	Organization (aggregate phenomenon)	Morrison and Milliken (2000); Milliken et al. (2003)
Perceived organizational silence climate (this study)	The degree to which an individual employee perceives that the belief that voice is meaningless or risky is prevalent within the organization (individual-level perception)	Individual (perception with an organizational referent)	Vakola and Bouradas (2005); Knoll and van Dick (2013)

These managerial practices operate through the immediate social context in which employees appraise whether voice is safe and useful. Perceptions of voice and silence are influenced by leadership, coworkers, and cultural context. Abusive supervision is related to silence through emotional exhaustion, and leader-member exchange can alter the strength of that relationship (Xu et al., 2015). Coworkers’ positive mood can promote voice under certain conditions (Liu et al., 2015), and voice norms vary across cultures (Kwon and Farndale, 2020). In addition, implicit voice beliefs, psychological safety, and voice efficacy are major conditions that explain whether employees speak up (Detert and Edmondson, 2011; Liang et al., 2012; Edmondson and Lei, 2014; Frazier et al., 2017). Research on silence regarding unethical practices also emphasizes the importance of the organizational environment (Harlos, 2016). Table 1 summarizes the differences in the level of analysis and the core focus of the related concepts.

### The perceived organizational silence climate, learned helplessness, psychological withdrawal, and job burnout

Learned helplessness is a cognitive state in which one believes that it is difficult to change outcomes through one’s own actions. Learned helplessness theory explains that when uncontrollable events are experienced repeatedly, expectations about the link between behavior and outcomes weaken, and motivation and behavioral willingness may subsequently decline even in controllable situations (Abramson et al., 1978). In organizations as well, continuous change and repeated experiences of frustration can increase learned helplessness and change fatigue (Chung et al., 2017), and the judgment that one’s actions are not reflected in outcomes can weaken the willingness to attempt improvement and to speak up. Employees who strongly perceive a silence climate appraise the likelihood that voice will produce change as low. In this case, silence can be regarded not as simple passivity but as a response that involves negative expectations about the link between behavior and outcomes. The belief that speaking up is unwise can lead to the abandonment of voice (Morrison and Milliken, 2000), and the process of remaining silent even after witnessing bullying has also been explained from the perspective of learned helplessness (Mazzone et al., 2024). This reasoning supports the following hypothesis.

Hypothesis 1. The perceived organizational silence climate will be positively associated with learned helplessness.

Psychological withdrawal is a state in which employees retract their interest in and emotional investment in their job and organization and maintain psychological distance in the performance of their role. This is theoretically grounded in personal disengagement, in which individuals separate themselves from role performance (Kahn, 1990), and in self-estrangement, in which individuals feel that work and the self have become separated (Golden and Veiga, 2018). Although it is adjacent to low job engagement (Saks, 2006; Rich et al., 2010), psychological withdrawal focuses on the withdrawal of interest and effort directed at the job and the organization. Meanwhile, work alienation encompasses meaninglessness and powerlessness with respect to work in general (Nair and Vohra, 2010), whereas the psychological withdrawal examined in this study focuses on the reduction of interest and effort. Accordingly, it was measured using the self-estrangement scale (Golden and Veiga, 2018), which asks whether respondents expect only a paycheck, feel like another cog in a machine at work, and reduce their interest in and effort toward their job. Psychological withdrawal is an internal distancing that precedes actual turnover, and it represents an early state of withdrawal in which employees remain in the organization but appraise the meaning of their job as low and exert only minimal effort. In such a state, discretionary effort and interaction with coworkers may also decrease. Self-estrangement undermined social exchange relationships with coworkers and job performance (Golden and Veiga, 2018), and repeated experiences of being ignored or treated without respect were related to work alienation through a decline in interpersonal trust (Xia et al., 2022). Employees who perceive the usefulness of voice to be low may reduce their psychological investment in their job and organization. When employees expect that raising problems will not be accepted or that those who speak up will suffer disadvantages, they may withdraw their expectations of organizational change and retain only the minimum effort required for role performance. Experiences in which problem-raising was not accepted are linked to a decrease in organizational involvement (Milliken et al., 2003), and the more acquiescent silence is perceived as routine, the greater the likelihood that psychological distancing will occur (Van Dyne et al., 2003). The mediating role of work alienation in the relationship between organizational silence and turnover intention has also been reported (Katıtaş, 2026). These findings support the following hypothesis.

Hypothesis 2. The perceived organizational silence climate will be positively associated with psychological withdrawal.

Job burnout is a state in which emotional resources have been depleted by sustained job demands. Maslach and Jackson (1981) presented emotional exhaustion, depersonalization, and reduced personal accomplishment as the core dimensions of job burnout, and subsequent research has shown that burnout is related to job demands and resources and to job attitudes (Maslach et al., 2001; Alarcon, 2011). Measurement instruments centered on emotional exhaustion have also been developed and adapted (Kristensen et al., 2005; Halbesleben and Demerouti, 2005; Sinval et al., 2019). Unlike temporary fatigue, when emotional exhaustion accumulates, work energy decreases and cynicism increases (Maslach et al., 2001). Meta-analyses have also shown that burnout is related to negative job outcomes such as turnover intention (Swider and Zimmerman, 2010). In an environment where voice is suppressed, repeated self-censorship and emotion suppression can consume employees’ cognitive and emotional resources. When employees cannot speak up even though they are aware of problems, work energy is directed toward emotion regulation, and this resource loss can be connected to burnout. Silence was uniquely related to burnout (Sherf et al., 2021), and longitudinal studies and meta-analyses have also confirmed the relationships of silence with emotional exhaustion and job burnout (Parker et al., 2025; Lainidi et al., 2026). Studies of teachers and platform work have also shown that silence or algorithmic working conditions were related to burnout (Kassandrinou et al., 2023; Dong et al., 2025). The following hypothesis was therefore proposed.

Hypothesis 3. The perceived organizational silence climate will be positively associated with job burnout.

### Learned helplessness, psychological withdrawal, job burnout, and turnover intention

Turnover intention is an employee’s intention to leave the current organization and a state of cognitive withdrawal that is formed prior to actual turnover. It is regarded as a key stage in the turnover decision-making process and as a representative antecedent that predicts actual turnover (Mobley, 1977; Tett and Meyer, 1993). When learned helplessness accumulates, employees may appraise the possibility of improving problems within the organization as low and may perceive turnover as an alternative for recovering a sense of control. Research on organizational justice tested learned helplessness as a mediator of the association between justice perceptions and turnover intention; however, neither the justice–helplessness nor the helplessness–turnover-intention path was statistically significant (Tayfur et al., 2013). In research on nurses, learned helplessness was associated with tenure intentions (Moreland et al., 2015). Recent research has reported that workplace hopelessness is positively related to turnover intention (Salazar-Altamirano et al., 2025). Although these three studies differ in context and in the scope of the concepts examined, they all show that the judgment that it is difficult to improve one’s work situation can weaken the willingness to remain in the organization. This evidence supports the following hypothesis.

Hypothesis 4. Learned helplessness will be positively associated with turnover intention.

Psychological withdrawal can be understood as an internal disengagement that appears prior to turnover. Psychological withdrawal, in which employees retract their expectations of change and exert only minimal effort, can be linked to the weakening of the exchange relationship between employees and the organization (Cropanzano and Mitchell, 2005; Golden and Veiga, 2018). Self-estrangement undermines social exchange relationships and job outcomes (Golden and Veiga, 2018), and work alienation mediated the relationship between organizational silence and turnover intention (Katıtaş, 2026). Psychological and behavioral withdrawal in the context of digital transformation was also related to turnover intention (Kim and Sohn, 2024). The following hypothesis was therefore proposed.

Hypothesis 5. Psychological withdrawal will be positively associated with turnover intention.

When job burnout is high, work energy decreases and remaining in the organization may be perceived as an additional loss of resources. Research from a job demands-resources perspective has shown that job burnout is related to health problems and turnover intention (Schaufeli and Bakker, 2004), and meta-analyses have also confirmed the association between burnout and turnover intention (Swider and Zimmerman, 2010). Recent studies have shown that workload and emotional demands, or destructive leadership, are connected to turnover intention through job burnout (Hung et al., 2025; Chen et al., 2025). Together, these findings support the following hypothesis.

Hypothesis 6. Job burnout will be positively associated with turnover intention.

### Indirect associations through learned helplessness, psychological withdrawal, and job burnout

Taken together, the preceding discussion suggests that the perceived organizational silence climate may be related to turnover intention through learned helplessness, psychological withdrawal, and job burnout. When employees perceive voice as meaningless or risky, they may judge that they cannot change the situation, withdraw their interest in and effort toward their job and organization, and consume emotional energy in the process of self-censorship. Rather than being mutually exclusive categories, these three variables primarily represent, respectively, a cognitive judgment about controllability, the withdrawal of psychological investment in the job and the organization, and the depletion of emotional resources (Abramson et al., 1978; Kahn, 1990; Maslach and Jackson, 1981). The focus of the principal response differs in this way. The three states are therefore specified as parallel pathways, and each model-implied indirect association is estimated while the other pathways are controlled, rather than being integrated into a single psychological state. Prior research shows that negative organizational environments can be connected to turnover intention through different psychological processes. Organizational silence was related to turnover intention through work alienation (Katıtaş, 2026), and workload and emotional demands were related to turnover intention through job burnout (Hung et al., 2025). These studies support the validity of the individual mediating pathways, but they did not consider learned helplessness, psychological withdrawal, and job burnout simultaneously in the relationship between the perceived organizational silence climate and turnover intention. In particular, there is relatively little direct empirical research on the learned helplessness pathway, and it therefore needs to be verified together with the other psychological states. Accordingly, this study specifies the three variables as parallel mediators and examines whether each indirect pathway emerges uniquely even after controlling for the associations of the other pathways. The preceding discussion supports the following hypotheses.

Hypothesis 7. The perceived organizational silence climate will be positively associated with turnover intention through learned helplessness.

Hypothesis 8. The perceived organizational silence climate will be positively associated with turnover intention through psychological withdrawal.

Hypothesis 9. The perceived organizational silence climate will be positively associated with turnover intention through job burnout.

## Materials and methods

### Research model

Drawing on the preceding literature and theoretical discussion, this study examines the relationships among the independent variable of the perceived organizational silence climate, the mediating variables of learned helplessness, psychological withdrawal, and job burnout, and the dependent variable of turnover intention. To test the associations of the perceived organizational silence climate with learned helplessness, psychological withdrawal, and job burnout, the associations of these three psychological states with turnover intention, and the indirect associations transmitted through the three variables, a research model based on structural equation modeling was constructed as shown in Figure 1.

**Figure 1:**
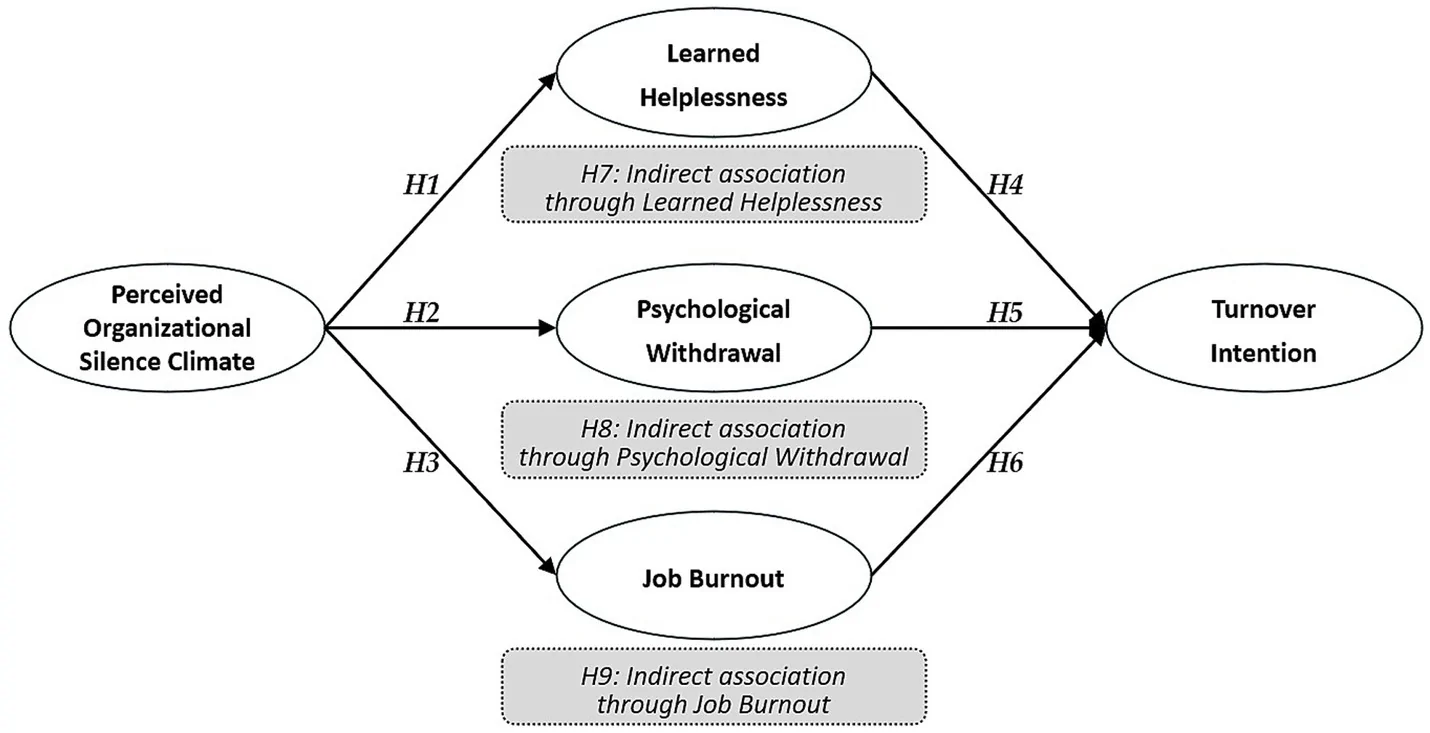
Research model. Solid arrows represent the six direct paths H1 to H6. The three shaded boxes with dotted borders identify the indirect-association hypotheses H7 to H9; each runs from the perceived organizational silence climate to turnover intention through the mediator shown immediately above the box. The direct path from the perceived organizational silence climate to turnover intention was estimated in the partial mediation model reported in Tables 4, 9 but is not drawn in the figure, because it is a non-hypothesized control path rather than a hypothesized relationship.

### Composition of the survey items

The survey items were constructed on the basis of prior research, as summarized in Table 2; the full wording of every item, in the original Korean administered to respondents and in English translation, is reported in Supplementary Table S1. The factors constituting the survey were operationally defined as follows. In this study, the perceived organizational silence climate refers to the degree to which employees perceive the belief that raising problems is useless or leads to disadvantage to be prevalent within the organization. It was measured with a focus on two elements: concerns about the disadvantages that follow the expression of dissenting opinions, and perceptions of supervisors’ unreceptive attitudes toward voice. Learned helplessness refers to a cognitive state in which the perception has become entrenched that outcomes cannot be controlled no matter how much effort is made to improve organizational problems, and it was measured with a focus on expectations about controllability and the abandonment of improvement attempts. Psychological withdrawal refers to a state in which employees perceive psychological distance between the meaning of their job and their own role and withdraw their emotions and interest from the job and the organization, and it was measured with a focus on the reduction of interest in the job and the decrease of effort. Job burnout refers to a state of emotional exhaustion caused by sustained emotional demands, and it was measured with a focus on fatigue and emotional depletion during work. Turnover intention refers to an employee’s intention to leave the current organization, and it was measured with a focus on plans to seek employment and consideration of resignation.

**Table 2:** Variables, number of measurement items, response scale, and source scales.

Variable	Number of items and response scale	Source scale
Perceived organizational silence climate	6 items; five-point Likert (1 = strongly disagree, 5 = strongly agree)	Vakola and Bouradas (2005)
Learned helplessness	4 items; five-point Likert (1 = strongly disagree, 5 = strongly agree)	Quinless and Nelson (1988); Chung et al. (2017)
Psychological withdrawal	5 items; five-point Likert (1 = strongly disagree, 5 = strongly agree)	Golden and Veiga (2018); Lehman and Simpson (1992)
Job burnout	4 items; five-point Likert (1 = strongly disagree, 5 = strongly agree)	Kristensen et al. (2005)
Turnover intention	3 items; five-point Likert (1 = strongly disagree, 5 = strongly agree)	Cammann et al. (1983)

Item codes correspond to those reported in Table 6. The complete item wording is provided in Supplementary Table S1.

The variables defined in this way were measured with a total of 22 survey items. The perceived organizational silence climate was measured with six items adapted from Vakola and Bouradas (2005). Three items on the expectation of disadvantage were taken from their top management attitudes to silence subscale, and three items on supervisors’ unreceptive attitudes toward voice were adapted, with reversed wording, from their supervisor’s attitudes to silence subscale. Learned helplessness was measured with four items adapted from the Learned Helplessness Scale developed by Quinless and Nelson (1988), which Chung et al. (2017) modified for the organizational context; these four items were then re-adapted to the digital platform service context of this study. Psychological withdrawal was measured with five items: four items were taken from the self-estrangement scale of Golden and Veiga (2018), and one item on reduced work effort was adapted from the psychological withdrawal behavior scale of Lehman and Simpson (1992). Job burnout was measured with four items adapted from the work-related burnout subscale of the Copenhagen Burnout Inventory (Kristensen et al., 2005); the interrogative frequency format of the original items was converted into declarative statements rated on the same five-point agreement scale used for the other constructs. Turnover intention was measured with three items constructed with reference to the turnover intention subscale of the Michigan Organizational Assessment Questionnaire (Cammann et al., 1983). All items were measured on a five-point Likert scale (1 = strongly disagree, 5 = strongly agree). The full wording of all items in the original Korean and in English translation is provided in Supplementary Table S1.

### Survey administration and analytical methods

This study conducted a survey of employees working at digital platform service firms in South Korea, covering information technology services, e-commerce, financial technology, and content platforms. The data were collected through a self-report online survey administered by a professional online research panel company from July 3 to July 13, 2026. Of the responses collected, 29 insincere responses were excluded, and a final total of 346 responses were used in the analysis.

The target population is employees of digital platform service firms in South Korea. This population is not enumerated in any official register, because Korean industrial statistics classify establishments by standard industry codes that do not isolate digital platform service providers as a separate category. A complete sampling frame was therefore unavailable and probability sampling was not feasible, so a non-probability purposive sample was drawn from the standing nationwide research panel maintained by the survey provider. Panel registration, identity management, and the prevention of duplicate participation are operated by the provider under its own quality-control procedures, and no personally identifiable information was transferred to the research team at any stage. The target population is defined by employer type, so eligibility was established at the beginning of the questionnaire through screening items on current employment status, organization type, and job area, and only respondents who reported working for a digital platform service firm in one of the four target sectors proceeded to the substantive items.

Invitations were distributed by the provider to its own member pool and the number of invitations issued was not reported to the research team, so a conventional response rate relative to the number of persons contacted cannot be computed. Of the 375 questionnaires that were completed and returned, 29 were excluded as insincere responses, giving a usable-response rate of 92.3% and a final analytic sample of 346. Insincerity was operationalized as patterned responding, that is, selecting an identical response option, or a fixed repeating pattern of options, across the questionnaire items. The sector composition of the final sample, together with the distribution of the remaining demographic characteristics, is reported in Table 3. Sample adequacy relative to the complexity of the estimated model is evaluated separately in the following subsection.

**Table 3:** Demographic characteristics of the survey respondents (*N* = 346).

Characteristic	Category	Frequency (n)	Percentage (%)
Gender	Male	198	57.2
	Female	148	42.8
Age	20s	92	26.6
	30s	151	43.6
	40s	78	22.5
	50s or older	25	7.2
Education	High school	13	3.8
	Junior college	42	12.1
	University	230	66.5
	Graduate school or higher	61	17.6
Tenure at current workplace	Less than 1 year	59	17.1
	1–3 years	116	33.5
	3–5 years	78	22.5
	5–10 years	65	18.8
	10 years or more	28	8.1
Organization type	IT services	111	32.1
	E-commerce	79	22.8
	Financial technology	56	16.2
	Content platform	71	20.5
	Other	29	8.4
Job area	Service/product planning/PM/design	77	22.3
	Development/engineering/data	105	30.3
	Sales/marketing/MD	62	17.9
	Customer support/operations	48	13.9
	Management support/administration	41	11.8
	Other	13	3.8

### Sample size and statistical power

The adequacy of the sample was evaluated in relation to model complexity rather than by a single universal cutoff. The 22 indicators yield 253 non-redundant elements in the observed variance–covariance matrix. The structural model freely estimates 54 parameters—17 factor loadings, 22 indicator error variances, the variance of the exogenous latent variable, 7 structural paths, 4 disturbance variances, and 3 disturbance covariances among the mediators—leaving 199 degrees of freedom. With 346 observations, the ratio of observations to freely estimated parameters is 6.4:1 and the ratio of observations to indicators is 15.7:1. These descriptive ratios are reported alongside model-based power because SEM sample-size requirements depend on model characteristics and the precision sought, rather than on a universal rule of thumb (Jackson, 2003; Wolf et al., 2013). Hoelter’s critical N was 327 at α = 0.05 and 348 at α = 0.01 (Hoelter, 1983).

Statistical power was evaluated with the RMSEA-based procedure of MacCallum et al. (1996). At 199 degrees of freedom and *N* = 346, power exceeded 0.999 both for the test of close fit (H_0_: RMSEA ≤ 0.05; H_1_: RMSEA = 0.08) and for the test of not-close fit (H_0_: RMSEA ≥ 0.08; H_1_: RMSEA = 0.05). The approximate sample sizes required for power of 0.80 under these two specifications are 85 and 86, respectively, after rounding up. These are omnibus tests of global model fit; they do not establish the precision of every structural path or of the pairwise contrasts between indirect associations. The latter are therefore interpreted from their bootstrap confidence intervals rather than from a claim that the sample can detect a universal minimum effect.

The distributional properties of the 22 indicators were also examined. Univariate skewness ranged from −0.333 to 0.158 and univariate excess kurtosis from −1.027 to −0.325, indicating modest item-level departures. Mardia’s coefficient of multivariate kurtosis was 510.540, compared with an expected value of 528.000 under multivariate normality, yielding a multivariate kurtosis value of −17.460 and a critical ratio of −4.997 (Supplementary Table S3). Thus, the multivariate normality test indicated a statistically detectable departure in the platykurtic direction. Multivariate non-normality can affect normal-theory CFA test statistics and standard errors (Curran et al., 1996). The maximum-likelihood fit results are therefore interpreted with appropriate caution; indirect associations and their pairwise contrasts were evaluated with 5,000-resample bias-corrected bootstrap confidence intervals.

Descriptive statistics and demographic characteristics were computed with IBM SPSS Statistics 28.0, and confirmatory factor analysis and structural equation modeling were conducted with maximum likelihood estimation in IBM SPSS Amos 28.0. Covariance-based structural equation modeling was retained because the study evaluates a confirmatory reflective common-factor model and relies on global model-fit information. Specific indirect-effect estimates are available in Amos, and pairwise contrasts among them can be estimated within the same covariance-based framework by applying bias-corrected bootstrapping to the contrast estimands; switching to partial least squares structural equation modeling was therefore unnecessary for the requested comparison. This choice also avoids changing the measurement-model estimand from common factors to composites (Rönkkö and Evermann, 2013; Hair et al., 2019).

The analysis proceeded in five steps. Following the two-step approach recommended by Anderson and Gerbing (1988), in which the measurement model is validated before the structural model is estimated (Kline, 2016; Hair et al., 2019), confirmatory factor analysis was used to examine the fit, reliability, and validity of the measurement model. The empirical distinctness of the three mediators was then examined by comparing the hypothesized five-factor measurement model with competing models in which the mediators were merged, using the changes in chi-square and descriptive fit indices. Multicollinearity among the predictors of turnover intention was assessed through variance inflation factors computed from both the latent correlation matrix and scale composites. Latent-variable structural equation modeling was then used to test the hypotheses. Finally, the specific indirect associations and the pairwise differences between them were estimated with bias-corrected bootstrap confidence intervals based on 5,000 resamples, following the multiple-mediator comparison procedure described by Preacher and Hayes (2008). The specific indirect associations reported in Table 4 were obtained as user-defined estimands in Amos. The pairwise differences reported in Table 5 were estimated from the same model by re-fitting it to 5,000 bootstrap resamples of the item-level data with an independent maximum-likelihood routine, because Amos does not report these contrasts by default. The same routine reproduced the Amos specific indirect associations, the direct association, and the total association to within Monte Carlo error, which confirms that the two implementations estimate the same model; both sets of results are reported side by side in Supplementary Table S4.

**Table 4:** Model-implied direct, specific indirect, and total associations.

Path	Model-implied association estimate (B)	95% BC CI
H7: Perceived organizational silence climate → learned helplessness → turnover intention	0.117	[0.072, 0.180]
H8: Perceived organizational silence climate → psychological withdrawal → turnover intention	0.192	[0.133, 0.275]
H9: Perceived organizational silence climate → job burnout → turnover intention	0.114	[0.072, 0.174]
Total indirect association	0.424	[0.329, 0.544]
Direct association (c′): Perceived organizational silence climate → turnover intention	0.046	[−0.069, 0.164]
Total association	0.470	[0.348, 0.607]

All values are unstandardized, model-implied association estimates from the partial mediation model that includes the direct path. BC CI denotes bias-corrected bootstrap confidence intervals based on 5,000 resamples. Total association = direct association + total indirect association. For the direct association (c′), S.E. = 0.058, C.R. = 0.803, and p = 0.422 under maximum likelihood estimation; the corresponding bootstrap two-tailed p value was 0.429. These decompositions are interpreted as associations, not causal effects.

**Table 5:** Pairwise comparisons of the specific indirect associations.

Contrast	Difference in indirect associations (B)	S.E.	95% BC CI	*p*
Psychological withdrawal pathway—learned helplessness pathway	0.075	0.044	[−0.008, 0.164]	0.075
Psychological withdrawal pathway—job burnout pathway	0.078	0.044	[−0.003, 0.169]	0.059
Learned helplessness pathway—job burnout pathway	0.003	0.035	[−0.062, 0.073]	0.944

All values are unstandardized. BC CI denotes bias-corrected bootstrap confidence intervals based on 5,000 resamples. S.E. is the bootstrap standard error of the difference and p is the bootstrap two-tailed probability. None of the three confidence intervals excludes zero.

## Results

### Demographic characteristics

As presented in Table 3, the gender composition of the respondents was 57.2% male and 42.8% female. With respect to age, respondents in their 30s were the most numerous at 43.6%, followed by those in their 20s at 26.6%, those in their 40s at 22.5%, and those aged 50 or older at 7.2%. In terms of education, university graduates accounted for the largest share at 66.5%, followed by those with a graduate degree or higher at 17.6%, junior college graduates at 12.1%, and high school graduates at 3.8%. With respect to tenure at the current workplace, 1 to 3 years was the most common at 33.5%. Organization type consisted of information technology services at 32.1%, e-commerce at 22.8%, content platforms at 20.5%, financial technology at 16.2%, and other at 8.4%. With respect to job area, development, engineering, and data was the most common at 30.3%, followed by service and product planning, project management, and design at 22.3%, sales, marketing, and merchandising at 17.9%, customer support and operations at 13.9%, management support and administration at 11.8%, and other at 3.8%. Taken together, the sample is concentrated among employees in their 20s and 30s (70.2%), respondents holding a university degree or higher (84.1%), and technical and planning functions (52.6% in service and product planning, project management, and design, or in development, engineering, and data), and half of the respondents (50.6%) had been with their current employer for less than 3 years. These concentrations describe the realized sample rather than the population distribution, because no complete sampling frame exists. Job area and tenure are therefore treated as substantive characteristics of this sample and as boundary conditions when the generalizability of the findings is considered.

### Reliability and validity

Table 6 presents the reliability and convergent validity results for the measurement model. The numbers of measurement items for each construct were six for the perceived organizational silence climate, four for learned helplessness, five for psychological withdrawal, four for job burnout, and three for turnover intention. The standardized factor loadings ranged from 0.708 to 0.940. Composite reliability ranged from 0.879 to 0.944, average variance extracted ranged from 0.548 to 0.813, and Cronbach’s α ranged from 0.879 to 0.943. All of these values exceed the conventional thresholds of 0.70 for composite reliability and Cronbach’s α, 0.50 for average variance extracted, and 0.70 for standardized factor loadings (Fornell and Larcker, 1981; Hair et al., 2019), and the results therefore support internal consistency and convergent validity. The critical ratios (C.R.) of the measurement items, excluding the reference item of each construct, were all statistically significant (*p* < 0.001). Analysis of the fit of the measurement model showed χ^2^(199) = 246.222 and χ^2^/df = 1.237. SRMR was 0.028, RMR was 0.033, GFI was 0.939, AGFI was 0.922, NFI was 0.959, TLI was 0.991, CFI was 0.992, and RMSEA was 0.026 (90% CI [0.013, 0.036]). These values meet the widely used cutoffs for the principal covariance-based SEM indices: CFI and TLI at or above 0.95, SRMR at or below 0.08, and RMSEA at or below 0.06 (Hu and Bentler, 1999; Kline, 2016). Eight complementary fit indices are reported, and the principal indices all meet their respective criteria, indicating excellent fit.

**Table 6:** Results of reliability and convergent validity testing.

Variable	Item	B	β	S.E.	C.R.	CR	AVE	α
Perceived organizational silence climate	POSC1	1.000	0.717	-	-	0.879	0.548	0.879
	POSC2	0.961	0.708	0.078	12.289***			
	POSC3	1.026	0.757	0.078	13.106***			
	POSC4	0.962	0.739	0.075	12.807***			
	POSC5	1.023	0.756	0.078	13.096***			
	POSC6	1.016	0.764	0.077	13.231***			
Learned helplessness	LH1	1.000	0.838	-	-	0.944	0.809	0.943
	LH2	1.076	0.930	0.046	23.388***			
	LH3	1.062	0.919	0.046	22.889***			
	LH4	1.057	0.908	0.047	22.435***			
Psychological withdrawal	PW1	1.000	0.766	-	-	0.902	0.648	0.901
	PW2	1.059	0.810	0.068	15.644***			
	PW3	1.124	0.855	0.068	16.639***			
	PW4	1.028	0.796	0.067	15.354***			
	PW5	1.063	0.796	0.069	15.352***			
Job burnout	JB1	1.000	0.821	-	-	0.939	0.795	0.938
	JB2	1.054	0.918	0.048	21.736***			
	JB3	1.040	0.922	0.048	21.894***			
	JB4	1.036	0.902	0.049	21.137***			
Turnover intention	TI1	1.000	0.850	-	-	0.929	0.813	0.927
	TI2	1.102	0.940	0.046	24.106***			
	TI3	1.030	0.912	0.045	23.046***			

B is the unstandardized factor loading of each item on its construct and β is the corresponding standardized factor loading; both are reported for every item. C.R. = critical ratio. The S.E. and C.R. of the reference item fixed at 1.000 for model identification are not computed. ***p < 0.001.

As presented in Table 7, discriminant validity was examined by applying both the Fornell-Larcker criterion and the HTMT criterion (Fornell and Larcker, 1981; Henseler et al., 2015). The square roots of the average variance extracted of each construct ranged from 0.740 to 0.902 and were all greater than the correlation coefficients between the corresponding construct and the other constructs, and the HTMT values between latent variables ranged from 0.317 to 0.609, falling below the criterion of 0.85, thereby securing discriminant validity. The highest HTMT value among the three mediators was 0.372.

**Table 7:** Results of discriminant validity testing.

Variable	AVE	(1)	(2)	(3)	(4)	(5)
(1) Perceived organizational silence climate	0.548	**0.740**	0.383	0.447	0.386	0.446
(2) Learned helplessness	0.809	0.373***	**0.899**	0.340	0.317	0.536
(3) Psychological withdrawal	0.648	0.445***	0.338***	**0.805**	0.372	0.609
(4) Job burnout	0.795	0.390***	0.318***	0.372***	**0.892**	0.524
(5) Turnover intention	0.813	0.427***	0.518***	0.606***	0.518***	**0.902**

The bold values on the diagonal are the square roots of AVE, the values below the diagonal are correlation coefficients among the latent variables, and the values above the diagonal are HTMT values. ***p < 0.001.

Learned helplessness, psychological withdrawal, and job burnout describe adjacent psychological states, and their empirical separability was therefore examined further by comparing the hypothesized five-factor measurement model with competing models in which the mediators were combined (Table 8). Merging learned helplessness with psychological withdrawal reduced CFI from 0.992 to 0.821; merging learned helplessness with job burnout reduced it to 0.781; and merging psychological withdrawal with job burnout reduced it to 0.827. The corresponding RMSEA values ranged from 0.119 to 0.134 and SRMR values from 0.139 to 0.149, compared with 0.026 and 0.028, respectively, for the hypothesized model. The model that combined all three mediators and the single-factor model also fit poorly. This consistent deterioration across several fit indices supports treating the three mediators as empirically distinguishable, although conceptually related, constructs.

**Table 8:** Comparison of the hypothesized measurement model with competing alternative models.

Model	χ^2^	df	CFI	TLI	RMSEA	SRMR	Δχ^2^	Δdf	ΔCFI
M1. Five-factor (hypothesized)	246.222	199	0.992	0.991	0.026	0.028	—	—	—
M2. Four-factor (LH and PW combined)	1237.465	203	0.821	0.796	0.122	0.149	991.243	4	0.171
M3. Four-factor (LH and JB combined)	1468.416	203	0.781	0.751	0.134	0.139	1222.194	4	0.211
M4. Four-factor (PW and JB combined)	1201.415	203	0.827	0.803	0.119	0.139	955.193	4	0.165
M5. Three-factor (LH, PW and JB combined)	2471.946	206	0.608	0.560	0.179	0.124	2225.724	7	0.384
M6. One-factor	3390.349	209	0.449	0.391	0.210	0.151	3144.126	10	0.543

LH, learned helplessness; PW, psychological withdrawal; JB, job burnout. All models were estimated by maximum likelihood on the same 22 indicators and 346 observations. Differences are reported descriptively relative to M1; no significance claim is based on the rounded differences.

Multicollinearity among the predictors of turnover intention was examined next. Variance inflation factors computed from the latent correlation matrix were 1.246 for learned helplessness, 1.361 for psychological withdrawal, 1.284 for job burnout, and 1.412 for the perceived organizational silence climate. The corresponding values computed from scale composites were 1.219, 1.293, 1.238, and 1.328. These uniformly low values indicate that the estimates are unlikely to be materially affected by collinearity among the parallel mediators.

### Common method bias

To examine the common method bias that may arise from measuring the independent and dependent variables through the same survey, procedural safeguards and statistical diagnostics were applied together (Podsakoff et al., 2003). The procedural remedies that were implemented consisted of guaranteeing the anonymity and confidentiality of responses, stating that participation was voluntary and informing respondents of the purpose of the research, and not collecting personally identifiable information. A temporal, spatial, or psychological separation of the predictor and criterion measures was not implemented, and all items were presented with an identical five-point Likert response format and a common response scale. In addition, a single common factor confirmatory factor analysis model was compared with the hypothesized five-factor measurement model. The fit of the single-factor model was very poor, with χ^2^(209) = 3390.349, χ^2^/df = 16.222, SRMR = 0.151, RMR = 0.175, GFI = 0.445, AGFI = 0.328, NFI = 0.436, TLI = 0.391, CFI = 0.449, and RMSEA = 0.210. Every incremental index of this model falls far below the conventional threshold of 0.90 and its RMSEA far exceeds 0.08 (Hu and Bentler, 1999; Kline, 2016). This is the expected outcome of the single-common-factor CFA: the deliberately constrained one-factor model should fit poorly if one factor cannot reproduce the covariance structure of the five constructs. By contrast, the fit of the five-factor measurement model was excellent, with SRMR = 0.028, CFI = 0.992, and RMSEA = 0.026. The poor fit of the single-factor model indicates that it is difficult to explain the covariances among the measures with a single common factor alone. However, this result alone cannot completely rule out common method bias, and it was therefore interpreted as limited evidence that common method bias is unlikely to have dominated the analytical results. The fit indices of all measurement and structural models are summarized in Supplementary Table S2.

### Hypothesis testing

All coefficients in Tables 4, 9 were derived from the partial mediation model that includes the direct path. This model freely estimates the disturbance covariances among the three mediating variables, so the structural portion is saturated and the model fit is identical to that of the measurement model. The fit of the structural model was χ^2^(199) = 246.222, χ^2^/df = 1.237, SRMR = 0.028, CFI = 0.992, and RMSEA = 0.026. The fit of the alternative full mediation model, which excludes the direct path, was χ^2^(200) = 246.864, χ^2^/df = 1.234, and SRMR = 0.029, and the chi-square difference from the partial mediation model was not significant (Δχ^2^(1) = 0.642, *p* = 0.423). The latent-variable structural equation model supported all six direct-association hypotheses, as shown in Table 9. The perceived organizational silence climate was positively associated with learned helplessness (β = 0.373, C.R. = 6.198, *p* < 0.001), psychological withdrawal (β = 0.445, C.R. = 7.007, *p* < 0.001), and job burnout (β = 0.390, C.R. = 6.429, *p* < 0.001). In addition, learned helplessness (β = 0.286, C.R. = 5.924, *p* < 0.001), psychological withdrawal (β = 0.392, C.R. = 7.156, *p* < 0.001), and job burnout (β = 0.266, C.R. = 5.424, *p* < 0.001) were positively associated with turnover intention, and Hypotheses 4 to 6 were therefore also supported. The direct association of the perceived organizational silence climate with turnover intention was not significant (β = 0.042, C.R. = 0.803, *p* = 0.422). The model explained 13.9% of the variance in learned helplessness, 19.8% of the variance in psychological withdrawal, 15.2% of the variance in job burnout, and 54.1% of the variance in turnover intention. Accordingly, the more strongly employees perceived the organizational silence climate, the higher their learned helplessness, psychological withdrawal, and job burnout were, and the higher these psychological states were, the higher their turnover intention was.

**Table 9:** Results of the structural model path estimates.

Hypothesis	Path	B	S.E.	C.R.	β	Result
H1	Perceived organizational silence climate → learned helplessness	0.398	0.064	6.198***	0.373	Supported
H2	Perceived organizational silence climate → psychological withdrawal	0.515	0.073	7.007***	0.445	Supported
H3	Perceived organizational silence climate → job burnout	0.403	0.063	6.429***	0.390	Supported
H4	Learned helplessness → turnover intention	0.295	0.050	5.924***	0.286	Supported
H5	Psychological withdrawal → turnover intention	0.373	0.052	7.156***	0.392	Supported
H6	Job burnout → turnover intention	0.283	0.052	5.424***	0.266	Supported

B = unstandardized coefficient; β = standardized coefficient. R^2^: learned helplessness = 0.139, psychological withdrawal = 0.198, job burnout = 0.152, turnover intention = 0.541. ***p < 0.001. C.R. = critical ratio.

### Indirect association analysis

The model-implied decomposition in Table 4 showed that all three indirect association estimates between the perceived organizational silence climate and turnover intention were statistically significant. The indirect association through learned helplessness was B = 0.117 (95% BC CI [0.072, 0.180]), the corresponding association through psychological withdrawal was B = 0.192 (95% BC CI [0.133, 0.275]), and the association through job burnout was B = 0.114 (95% BC CI [0.072, 0.174]), and Hypotheses 7 to 9 were therefore all supported. The total indirect association was B = 0.424 (95% BC CI [0.329, 0.544]) and the total association was B = 0.470 (95% BC CI [0.348, 0.607]), whereas the direct association after controlling for the mediating variables was not significant (B = 0.046, S.E. = 0.058, C.R. = 0.803, *p* = 0.422, 95% BC CI [−0.069, 0.164]). This pattern indicates that the perceived organizational silence climate is related to turnover intention through learned helplessness, psychological withdrawal, and job burnout rather than being directly related to it. The three mediators were specified as parallel pathways, and the relative magnitudes of their specific indirect association estimates were therefore compared directly rather than inferred from the relative size of the coefficients. Each pairwise difference was tested with bias-corrected bootstrap confidence intervals based on 5,000 resamples, and the results are reported in Table 5. The difference between the psychological withdrawal pathway and the learned helplessness pathway was B = 0.075 (95% BC CI [−0.008, 0.164]), the difference between the psychological withdrawal pathway and the job burnout pathway was B = 0.078 (95% BC CI [−0.003, 0.169]), and the difference between the learned helplessness pathway and the job burnout pathway was B = 0.003 (95% BC CI [−0.062, 0.073]). None of the three confidence intervals excluded zero. The indirect association through psychological withdrawal was therefore numerically the largest, but the three pathways cannot be distinguished in magnitude at conventional levels of significance, and no pathway is identified as dominant.

## Discussion

This study analyzed the parallel indirect associations of learned helplessness, psychological withdrawal, and job burnout in the relationship between the perceived organizational silence climate and turnover intention among members of digital platform service organizations. The results showed that the perceived organizational silence climate was positively associated with the three psychological states, and that all three psychological states were also positively associated with turnover intention. Three findings are noteworthy.

First, employees who perceived a stronger organizational silence climate reported higher learned helplessness, psychological withdrawal, and job burnout. Specifically, employees who appraise the usefulness of voice and the controllability of outcomes as low experience greater learned helplessness, stronger self-estrangement, and more severe emotional exhaustion. These findings have implications similar to those of Sherf et al. (2021), who confirmed the unique association between silence and job burnout; Parker et al. (2025), who demonstrated the concurrent association between silence and emotional exhaustion; and studies that explained environments in which voice is suppressed from the perspective of learned helplessness (Mazzone et al., 2024; Chung et al., 2017). The result shows that silence needs to be interpreted not only as an individual’s passive attitude but also as a psychological demand related to the perception of the organizational environment. In particular, the finding that the three psychological states were simultaneously elevated indicates that the perception of an organizational silence climate can be broadly related to employees’ judgments, their psychological investment in the job and the organization, and their emotional energy. Learned helplessness solidifies the judgment that it is difficult to change the situation through voice, psychological withdrawal reduces interest and effort, and job burnout weakens the emotional resources needed to sustain work. Digital platform service organizations continuously adjust their operating methods in response to user reactions and technological change, and the sharing of frontline information is therefore important (Nambisan et al., 2017; de Reuver et al., 2018).

Second, learned helplessness, psychological withdrawal, and job burnout were all positively associated with turnover intention. The indirect association through psychological withdrawal was numerically the largest, although the pairwise comparisons reported in Table 5 show that the three specific indirect associations do not differ significantly in magnitude, so this ordering should not be read as evidence that one pathway predominates. One reading of the pattern is that the withdrawal of psychological investment in the job and the organization accompanies the formation of turnover intention; because all variables were measured at a single point in time, however, the temporal ordering of these states cannot be established from the present data. The finding of Katıtaş (2026) that organizational silence is associated with turnover intention through work alienation and the finding of Golden and Veiga (2018) that self-estrangement weakens social exchange relationships with coworkers support this interpretation. Accordingly, organizations need to identify signals of turnover not at the point of resignation but at the earlier stage of reduced involvement. The result indicates that turnover management should not remain limited to directly ascertaining employees’ intention to resign. Psychological withdrawal, such as decreased interest in work, minimal role performance, and indifference toward organizational problems, may co-occur with and could plausibly precede a formal intention to leave, but that sequence requires longitudinal verification. Therefore, organizations need to regularly examine changes in employees’ level of participation and sense of work meaningfulness, in addition to *post hoc* indicators such as turnover rates and exit interviews.

Third, the direct association between the perceived organizational silence climate and turnover intention was not significant once the three mediators were included. This pattern is consistent with an account in which the relationship between the perception of a silence climate and turnover intention operates through psychological depletion rather than directly. The design is cross-sectional, so this is an account with which the data are consistent rather than a mechanism the data establish. This result is consistent with the logic of the health impairment pathway of job demands-resources theory, according to which job demands lead to negative outcomes through a process of psychological depletion (Schaufeli and Bakker, 2004), and with the logic of conservation of resources theory, according to which the loss of resources leads to strain and withdrawal (Hobfoll, 1989). In addition, compared with prior studies that tested a single mediating pathway (Katıtaş, 2026; Hung et al., 2025; Cicek et al., 2021), this study is distinctive in that it verified the three pathways of cognitive judgment, withdrawal of psychological investment, and emotional exhaustion together within a single model, thereby describing the relationship between silence and turnover more precisely. The finding that the direct association was not significant while all three indirect association estimates were significant suggests that employees’ psychological states need to be considered together in the process through which a silence climate is connected to turnover intention. In other words, measures to alleviate the learned helplessness, psychological withdrawal, and job burnout that have already formed must be implemented in parallel with efforts to improve the voice environment. Managing only one of the three pathways may allow turnover intention to increase through the other psychological pathways. It is therefore necessary to design voice systems, managers’ receptive leadership behaviors, the expansion of job resources, and recovery support in an integrated manner.

## Conclusion

### Implications

This study empirically analyzed the association between the perceived organizational silence climate and turnover intention among members of digital platform service organizations. This study makes three theoretical contributions. First, this study expanded the scope of organizational silence research by presenting employees’ perception of the organizational silence climate, rather than individual silence behavior, as an organizational contextual correlate of turnover intention, and by empirically examining that relationship. Whereas silence research to date has concentrated on why employees remain silent, this study addressed how employees perceive an environment in which silence has become routine and what outcomes are associated with that perception. Second, this study distinguished three pathways centered on the cognitive judgment of controllability in learned helplessness, the withdrawal of psychological investment in psychological withdrawal, and the emotional exhaustion of job burnout, and estimated the indirect associations of the three pathways together within a single model, establishing both that the three constructs are empirically distinguishable and that their specific indirect associations do not differ significantly in magnitude. Third, this study interpreted the perceived organizational silence climate as a job demand that induces psychological burden, thereby applying the health impairment pathway of job demands-resources theory to the silence literature, and connected this to conservation of resources theory by interpreting the resources that may be weakened in this process from the perspectives of a sense of control, relational investment, and emotional energy.

The findings also offer practical implications. First, in turnover management, organizations should regularly examine not only compensation and career development but also employees’ perceptions of the usefulness and safety of voice. Organizations should establish formal upward communication channels and anonymous reporting channels through which employees can raise problems and operate feedback procedures that inform employees of the results of review and the follow-up measures taken in response to the opinions received. It is more important to provide the experience that opinions are actually being reviewed than merely to provide the existence of channels. This is because employees may come to believe that speaking up again is useless if nothing changes after they speak up or if the handling process is unclear. This direction is also consistent with the results of Sherf et al. (2021), who confirmed that silence is uniquely related to low psychological safety and low perceived impact.

Second, to improve the voice environment, managers’ receptive behaviors and procedures for preventing disadvantage must be established together. Managers should treat dissenting opinions as information for improving work rather than accepting them as challenges to themselves personally and should operate procedures for ascertaining minority opinions in meetings and decision-making processes. In addition, it is necessary to clearly disclose evaluation criteria and processes and procedures for raising objections, and to institutionalize protective principles so that raising problems or asking questions does not lead to disadvantages in personnel decisions.

Third, organizations should not regard turnover intention solely as a problem that arises immediately before resignation, but should identify changes in learned helplessness, psychological withdrawal, and job burnout at an early stage. Through regular organizational diagnoses or short-cycle surveys, organizations can jointly examine the perception that nothing changes even when opinions are raised, the decrease of interest in and effort toward the job, and sustained emotional fatigue. For employees in whom psychological withdrawal is identified, organizations should consider interviews that reaffirm work meaningfulness and roles, the expansion of opportunities for participation, and work redesign; and for organizations in which job burnout is high, workload adjustment and support for rest and recovery should be implemented in parallel. Combining improvements to the voice environment with support for employees’ psychological states can help organizations address turnover risk earlier.

### Limitations and directions for future research

This study has four limitations. First, the sample was limited to employees of digital platform service firms in South Korea, which constrains generalizability across industries, regions, and cultures. The study did not directly compare platform with non-platform organizations, so it cannot establish whether the reported associations are specific to or stronger in platform settings. Future studies should examine differences in voice norms, organizational characteristics, and cultural context using samples from manufacturing, public institutions, traditional services, and multiple countries. A matched platform/non-platform design using multigroup structural equation modeling could directly test path equality and whether platform features such as algorithmic management and rapid iteration cycles moderate the three pathways.

Second, all variables were collected from the same respondents at a single point in time. Although comparison with a single common factor indicates that common method bias is unlikely to have dominated the results, cross-sectional data cannot establish temporal ordering, and the reported associations should not be interpreted causally. Single-wave mediation estimates can be biased relative to corresponding longitudinal indirect estimates even when the assumed causal structure is correct; the direction and magnitude depend on construct stability and the intervals over which the processes unfold (Cole and Maxwell, 2003; Maxwell and Cole, 2007; Maxwell et al., 2011). Causal interpretation also requires no unmeasured mediator-outcome confounding, an assumption this design cannot test (Rohrer et al., 2022). The estimates are therefore statistical decompositions under the specified model, not evidence of causal transmission. Future research should use time-lagged, half-longitudinal, full three-wave, or multi-source designs and examine leadership, organizational support, and algorithmic transparency as boundary conditions.

Third, the perceived organizational silence climate was assessed as an individual-level psychological climate perception. Respondents came from a nationwide panel without organizational identifiers, so *r*_wg_, ICC(1), and ICC(2) cannot be computed and no inference is made about whether the perceptions are shared at the organizational level. Establishing climate as a shared property requires multilevel samples of employees nested within identified units. Such research should test whether the individual-level associations also hold when climate is modeled as an aggregate unit-level property. That design should distinguish within-unit agreement from individual perception.

Fourth, although the three mediators were empirically distinguishable and each specific indirect association was statistically significant, none of the pairwise contrasts was significant. These null contrasts do not demonstrate that the indirect associations are equal; their confidence intervals remain compatible with both small and potentially meaningful differences. Studies designed to rank the pathways should prespecify the smallest substantively important contrast and determine sample size by Monte Carlo simulation for the planned latent-variable model. Such contrasts may require samples larger than those needed for global fit.

## Data Availability

The raw data supporting the conclusions of this article will be made available by the authors, without undue reservation.

## Supplementary material

Supplementary Tables S1–S4 (S1) Full measurement instrument in the original Korean with English translations; (S2) fit indices of all measurement and structural models; (S3) univariate and multivariate normality diagnostics for the 22 measurement items; (S4) specific indirect associations, the direct association, and pairwise contrasts from the Amos estimands and the independent bootstrap replication.
