# Supplementary material for: Perceived organizational silence climate and turnover intention: indirect associations through learned helplessness, psychological withdrawal, and job burnout
Source: Front Psychol. 2026 Oct 5;17:1962689. doi: 10.3389/fpsyg.2026.1962689 (PMC13677457; doi:10.3389/fpsyg.2026.1962689)
Supplement: Supplementary file 1 [file Table_1.docx]

Supplementary Material

# 1 Supplementary Tables

This Supplementary Material provides (i) the full wording of all measurement items in both the original Korean administered to respondents and the English translation used in the manuscript, (ii) the fit indices of all measurement and structural models reported in the manuscript, (iii) the univariate and multivariate normality diagnostics for the 22 measurement items, and (iv) the full bootstrap output for the specific indirect associations and their pairwise contrasts. No data beyond those reported in the main manuscript are presented.

**Supplementary Table S1. Full measurement instrument: original Korean items and English translations.**

| **Construct** | **Item code** | **Original Korean item (as administered)** | **English translation** |
| --- | --- | --- | --- |
| Perceived organizational silence climate | POSC1 | 회사 의견에 반대하면 문제를 일으키는 사람으로 여겨질 수 있다. | If you disagree about company issues, you can be characterised as a troublemaker. |
|  | POSC2 | 회사 의견에 반대 의견을 표현하면 부정적 일을 겪을 수 있다. | If you express your disagreements regarding company issues, you may suffer negative consequences. |
|  | POSC3 | 회사 의견에 반대하면 충성심 부족으로 인식된다. | If you disagree about company issues, it can be perceived as lack of loyalty. |
|  | POSC4 | 나의 상사는 구성원들이 말하는 내용에 주의를 기울이지 않는다. | My supervisor does not pay attention to what employees say. |
|  | POSC5 | 나의 상사는 구성원들이 서로 다른 의견이나 반대 의견을 표현하도록 장려하지 않는다. | My supervisor does not encourage employees to express different or dissenting opinions. |
|  | POSC6 | 나의 상사는 구성원들 사이의 갈등을 잘 처리하지 않는다. | My supervisor does not handle conflicts among employees well. |
| Learned helplessness | LH1 | 나는 조직의 문제를 개선하기 위해 아무리 노력해도, 그 결과를 통제할 수 없다고 느낀다. | I feel that no matter how hard I try to improve organizational problems, I cannot control the outcome. |
|  | LH2 | 나는 조직 내 주요 문제를 해결할 수 있는 능력이 부족하다고 느낀다. | I feel that I lack the ability to solve major problems within the organization. |
|  | LH3 | 나는 과거에 의견 제기나 개선 시도가 받아들여지지 않은 경험이 있으면, 이후 새로운 의견 제기를 시도하지 않는다. | If my opinions or improvement attempts were not accepted in the past, I do not attempt to raise new opinions afterward. |
|  | LH4 | 나는 조직을 개선하기 위해 의견을 내거나 노력해도 실제 변화에는 큰 영향을 미치지 못한다고 느낀다. | I feel that even if I offer opinions or make efforts to improve the organization, they do not have much effect on actual change. |
| Psychological withdrawal | PW1 | 내가 일에서 기대하는 유일한 것은 급여를 받는 것이다. | The only thing I look forward to in my work is getting paid. |
|  | PW2 | 일할 때 시간이 정말 느리게 간다. | Time really drags for me when I'm at work. |
|  | PW3 | 일할 때 나는 기계의 또 다른 부품이 된 것처럼 느낀다. | When I'm working, I feel like I've become just another part of a machine. |
|  | PW4 | 예전에는 내 업무 부서에 관심이 있었지만 이제는 그렇지 않다. | I used to care more about my work unit, but not anymore. |
|  | PW5 | 나는 마땅히 해야 할 것보다 직무에 더 적은 노력을 기울인다. | I put less effort into my job than I should have. |
| Job burnout | JB1 | 나는 또 하루 일하러 가야 한다고 생각하면 아침부터 지쳐 있다고 느낀다. | I am exhausted in the morning at the thought of another day at work. |
|  | JB2 | 나는 근무 시간 내내 피곤하다고 느낀다. | I feel that every working hour is tiring for me. |
|  | JB3 | 나의 일은 정서적으로 나를 지치게 한다. | My work is emotionally exhausting. |
|  | JB4 | 나는 내 일 때문에 소진되었다고 느낀다. | I feel burnt out because of my work. |
| Turnover intention | TI1 | 나는 내년에 새로운 직장을 찾을 가능성이 높다. | I will probably look for a new job in the next year. |
|  | TI2 | 나는 퇴사를 자주 생각한다. | I often think about quitting. |
|  | TI3 | 내년에 새로운 직장을 적극적으로 찾아볼 생각이다. | I intend to actively search for a new job within the next year. |

All items were measured on a five-point Likert scale (1 = strongly disagree, 5 = strongly agree). Item codes correspond to those reported in Table 6 of the main manuscript. Source scales: perceived organizational silence climate, Vakola and Bouradas (2005); learned helplessness, Quinless and Nelson (1988) as modified by Chung et al. (2017); psychological withdrawal, Golden and Veiga (2018) for PW1–PW4 and Lehman and Simpson (1992) for PW5; job burnout, Kristensen et al. (2005); turnover intention, Cammann et al. (1983).

**Supplementary Table S2. Fit indices of the measurement and structural models.**

| **Fit index** | **Measurement model (hypothesized five-factor model)** | **Single common factor model** | **Structural model A: partial mediation (direct path included)** | **Structural model B: full mediation (direct path excluded)** |
| --- | --- | --- | --- | --- |
| χ² | 246.222 | 3390.349 | 246.222 | 246.864 |
| df | 199 | 209 | 199 | 200 |
| χ²/df | 1.237 | 16.222 | 1.237 | 1.234 |
| SRMR | 0.028 | 0.151 | 0.028 | 0.029 |
| RMR | 0.033 | 0.175 | 0.033 | 0.034 |
| GFI | 0.939 | 0.445 | 0.939 | 0.939 |
| AGFI | 0.922 | 0.328 | 0.922 | 0.922 |
| NFI | 0.959 | 0.436 | 0.959 | 0.959 |
| TLI | 0.991 | 0.391 | 0.991 | 0.991 |
| CFI | 0.992 | 0.449 | 0.992 | 0.992 |
| RMSEA | 0.026 | 0.210 | 0.026 | 0.026 |

N = 346. The 90% confidence interval of RMSEA for the measurement model was [0.013, 0.036]. The chi-square difference between Structural model A and Structural model B was not significant (Δχ²(1) = 0.642, p = 0.423). Structural model A freely estimates the disturbance covariances among the three mediators, so its structural portion is saturated and its fit is identical to that of the measurement model. All values were taken from the IBM SPSS Amos 28.0 output for the corresponding model.

**Supplementary Table S3. Univariate and multivariate normality diagnostics for the 22 measurement items (N = 346).**

| **Item** | **M** | **SD** | **Skewness** | **Kurtosis** |
| --- | --- | --- | --- | --- |
| POSC1 | 2.971 | 1.137 | −0.026 | −0.739 |
| POSC2 | 2.850 | 1.106 | 0.158 | −0.669 |
| POSC3 | 2.893 | 1.105 | 0.070 | −0.622 |
| POSC4 | 2.968 | 1.061 | 0.005 | −0.509 |
| POSC5 | 2.991 | 1.102 | 0.017 | −0.748 |
| POSC6 | 2.902 | 1.083 | 0.141 | −0.606 |
| LH1 | 2.896 | 1.036 | 0.100 | −0.523 |
| LH2 | 2.873 | 1.005 | 0.033 | −0.452 |
| LH3 | 2.844 | 1.004 | 0.058 | −0.325 |
| LH4 | 2.850 | 1.010 | 0.051 | −0.380 |
| PW1 | 3.121 | 1.231 | −0.139 | −0.935 |
| PW2 | 3.240 | 1.234 | −0.176 | −0.974 |
| PW3 | 3.240 | 1.241 | −0.162 | −0.905 |
| PW4 | 3.220 | 1.217 | −0.049 | −0.950 |
| PW5 | 3.127 | 1.258 | −0.040 | −1.027 |
| JB1 | 3.350 | 1.025 | −0.255 | −0.460 |
| JB2 | 3.327 | 0.966 | −0.189 | −0.395 |
| JB3 | 3.263 | 0.950 | −0.180 | −0.343 |
| JB4 | 3.283 | 0.966 | −0.128 | −0.357 |
| TI1 | 3.483 | 1.056 | −0.333 | −0.544 |
| TI2 | 3.454 | 1.052 | −0.283 | −0.560 |
| TI3 | 3.509 | 1.014 | −0.276 | −0.481 |

N = 346. M and SD are the item mean and standard deviation. Skewness and kurtosis are the sample estimators reported by IBM SPSS Statistics 28.0; kurtosis is expressed as excess kurtosis. Univariate skewness ranged from −0.333 to 0.158 and univariate excess kurtosis from −1.027 to −0.325. Mardia's coefficient of multivariate kurtosis was 510.540, compared with an expected value of 528.000 under multivariate normality, yielding a multivariate kurtosis value of −17.460 and a critical ratio of −4.997. The critical ratio indicates a statistically detectable departure from multivariate normality in the platykurtic direction. Multivariate non-normality can affect normal-theory CFA test statistics and standard errors (Curran et al., 1996), so the maximum-likelihood results should be interpreted with appropriate caution. The indirect associations and their pairwise contrasts were evaluated with bias-corrected bootstrap confidence intervals based on 5,000 resamples.

**Supplementary Table S4. Specific indirect associations, the direct association, and pairwise contrasts: Amos user-defined estimands and independent bootstrap replication (N = 346, 5,000 bias-corrected resamples).**

| **Quantity** | **Amos B** | **Amos 95% BC CI** | **Replication B** | **Replication S.E.** | **Replication 95% BC CI** | **Replication bootstrap p** |
| --- | --- | --- | --- | --- | --- | --- |
| Indirect association via learned helplessness | 0.117 | [0.072, 0.180] | 0.117 | 0.027 | [0.072, 0.178] | < 0.001 |
| Indirect association via psychological withdrawal | 0.192 | [0.133, 0.275] | 0.192 | 0.036 | [0.133, 0.272] | < 0.001 |
| Indirect association via job burnout | 0.114 | [0.072, 0.174] | 0.114 | 0.025 | [0.071, 0.169] | < 0.001 |
| Total indirect association | 0.424 | [0.329, 0.544] | 0.424 | 0.054 | [0.327, 0.535] | < 0.001 |
| Direct association (c′) | 0.046 | [-0.069, 0.164] | 0.046 | 0.057 | [-0.066, 0.156] | 0.439 |
| Total association | 0.470 | [0.348, 0.607] | 0.470 | 0.067 | [0.340, 0.599] | < 0.001 |
| Difference in indirect associations: psychological withdrawal − learned helplessness | – | – | 0.075 | 0.044 | [-0.008, 0.164] | 0.075 |
| Difference in indirect associations: psychological withdrawal − job burnout | – | – | 0.078 | 0.044 | [-0.003, 0.169] | 0.059 |
| Difference in indirect associations: learned helplessness − job burnout | – | – | 0.003 | 0.035 | [-0.062, 0.073] | 0.944 |

All values are unstandardized, model-implied association estimates from the partial mediation model that includes the direct path. The Amos columns reproduce the user-defined estimands and bias-corrected bootstrap intervals reported in Table 4 of the main manuscript. The replication columns were obtained by re-fitting the same model to 5,000 bootstrap resamples of the item-level data with an independent maximum-likelihood routine, from which the bias-corrected percentile intervals and two-tailed bootstrap probabilities were computed; the contrasts reported in Table 5 of the main manuscript come from this replication, because Amos does not report pairwise differences between specific indirect associations by default. The two implementations agree on every quantity that both produce, to within the Monte Carlo error of a 5,000-resample bootstrap, which confirms that they estimate the same model. A dash indicates a quantity that Amos does not report by default.
